# Treatment of medulloblastoma using an oncolytic measles virus encoding the thyroidal sodium iodide symporter shows enhanced efficacy with radioiodine

**DOI:** 10.1186/1471-2407-12-508

**Published:** 2012-11-07

**Authors:** Brian Hutzen, Christopher R Pierson, Stephen J Russell, Evanthia Galanis, Corey Raffel, Adam W Studebaker

**Affiliations:** 1 Molecular, Cellular, and Developmental Biology Program, The Ohio State University, Columbus, OH, 43205, USA; 2 Department of Pathology, The Ohio State University College of Medicine, Columbus, OH, 43210, USA; 3 Department of Molecular Medicine, Mayo Clinic, Rochester, MN, 55905, USA; 4 Division of Medical Oncology, Mayo Clinic, Rochester, MN, 55905, USA; 5 Department of Neurological Surgery, The Ohio State University College of Medicine, Columbus, OH, 43210, USA; 6 The Center for Childhood Cancer, The Research Institute at Nationwide Children's Hospital, Columbus, Ohio, 43205, USA

**Keywords:** Medulloblastoma, Measles virus, Sodium iodide symporter, Targeted radiotherapy

## Abstract

**Background:**

Medulloblastoma is the most common malignant brain tumor of childhood. Although the clinical outcome for medulloblastoma patients has improved significantly, children afflicted with the disease frequently suffer from debilitating side effects related to the aggressive nature of currently available therapy. Alternative means for treating medulloblastoma are desperately needed. We have previously shown that oncolytic measles virus (MV) can selectively target and destroy medulloblastoma tumor cells in localized and disseminated models of the disease. MV-NIS, an oncolytic measles virus that encodes the human thyroidal sodium iodide symporter (NIS), has the potential to deliver targeted radiotherapy to the tumor site and promote a localized bystander effect above and beyond that achieved by MV alone.

**Methods:**

We evaluated the efficacy of MV-NIS against medulloblastoma cells *in vitro* and examined their ability to incorporate radioiodine at various timepoints, finding peak uptake at 48 hours post infection. The effects of MV-NIS were also evaluated in mouse xenograft models of localized and disseminated medulloblastoma. Athymic nude mice were injected with D283med-Luc medulloblastoma cells in the caudate putamen (localized disease) or right lateral ventricle (disseminated disease) and subsequently treated with MV-NIS. Subsets of these mice were given a dose of ^131^I at 24, 48 or 72 hours later.

**Results:**

MV-NIS treatment, both by itself and in combination with ^131^I, elicited tumor stabilization and regression in the treated mice and significantly extended their survival times. Mice given ^131^I were found to concentrate radioiodine at the site of their tumor implantations. In addition, mice with localized tumors that were given ^131^I either 24 or 48 hours after MV-NIS treatment exhibited a significant survival advantage over mice given MV-NIS alone.

**Conclusions:**

These data suggest MV-NIS plus radioiodine may be a potentially useful therapy for the treatment of medulloblastoma.

## Background

Medulloblastoma is the most common malignant brain tumor of childhood [1]. Our understanding of this disease, its etiology, and treatment has improved considerably over the past several years and is reflected in 5-year survival rates that now exceed 70% [2]. Despite these advancements, numerous challenges in the effective treatment of medulloblastoma remain. Conventional therapy, consisting of surgical resection and craniospinal irradiation with or without chemotherapy, is frequently associated with neurocognitive morbidity. Patients treated for medulloblastoma often display impaired intelligence and deficits in processing speed, memory ability, and attention, which significantly impact their quality of life [3,4]. In addition, a sizable subset of medulloblastoma patients will effectively remain incurable, owing to medulloblastoma’s propensity to disseminate in cerebrospinal fluid (CSF) spaces, including the ventricles, intracranial subarachnoid space, and spinal subarachnoid space [5,6]. Fewer than 20% of children who present with disseminated medulloblastoma will survive more than five years [7]. Alternative treatment modalities for medulloblastoma are clearly needed.

One promising approach is the development and use of oncolytic measles viruses (MV). As derivatives of the Edmonston vaccine strain, these viruses display a natural tropism for the CD46 membrane protein, an inhibitory complement regulator strongly over-expressed by many types of tumor relative to normal tissue [8,9]. MV preferentially infects tumor cells and induces their death via syncytia formation and apoptosis, causing minimal damage to the normal surrounding tissue [10,11]. In a recently published study, we reported that the majority of medulloblastomas over-express CD46 and were consequently susceptible to MV oncolysis [12]. We also demonstrated that MV virotherapy was effective against orthotopic mouse xenograft models of localized and disseminated medulloblastoma [12,13]. In these studies, multiple intratumoral injections of MV were found to significantly reduce tumor burden and extend survival in treated animals. Further studies aimed at scaling back the number of intratumoral MV injections revealed a marked decrease in the efficacy of the treatment (unpublished data), prompting us to explore the use of genetically modified MV that offer enhanced killing of tumor cells.

The insertion of specific transgenes into the MV genome can be used to confer increased specificity, augment MV killing of infected tumor cells, or provide markers to assess virus delivery and tumor response [9]. MV-NIS, an oncolytic MV engineered to express the human thyroidal sodium iodide symporter (NIS), was developed to provide a noninvasive means of imaging tumors and to potentially enhance the efficacy of MV against radiosensitive malignancies by concentrating radioiodine in virus-infected cells [14]. MV-NIS was shown to exhibit a profound synergy with the β^-^ particle emitting radioiodine isotope ^131^I in a multiple myeloma xenograft model, wherein the administration of 37 MBq ^131^I at peak infection resulted in complete tumor regression in all the animals under study [14]. More recently, the combination of MV-NIS and ^131^I was found to have significant antitumor activity against an orthotopic model of glioblastoma multiforme, an invasive and radiosensitive primary brain tumor [15]. Because medulloblastomas are also known to be extremely radiosensitive [2], we hypothesized that MV-NIS virotherapy in combination with ^131^I could promote enhanced tumor regression and survival in our orthotopic models of localized and disseminated disease.

## Methods

### Cell culture

The Vero, D283med and UW426 cell lines were obtained from the American Type Culture Collection. The D283med-Luc cell line was generated as described previously [12]. These cell lines were maintained in DMEM supplemented with 10-20% FBS, 1% penicillin/streptomycin and 2mM L-glutamine and cultured at 37°C in a humidified incubator set at 5% CO_2_. The FRTL-5 cell line was the kind gift of Dr. Lawrence Kirschner of the Ohio State University and was cultured as described elsewhere [16].

### MV-NIS production and titration

The MV-NIS virus was the kind gift of Stephen J. Russell at the Mayo Clinic in Rochester, Minnesota. MV-NIS stocks were propagated by infecting Vero cells at an MOI of 0.01 in a minimal volume of OptiMEM (Invitrogen, Carlsbad, CA) for 2 hours. Unbound virus was then removed and replaced with DMEM with 10% FBS and the cells were incubated an additional 48–72 hours at 37°C. When the majority of the Vero cells had fused into syncytia, the media was removed and the cells were scraped into a small volume of OptiMEM. MV-NIS was harvested by two cycles of freezing in liquid nitrogen and thawing, followed by centrifugation at 10,000xG to pellet and remove cellular debris. Aliquoted virus was stored at −80°C. Viral titers were determined by 50% tissue culture infective dose (TCID_50_) titration on Vero cells [17].

### In vitro infection assays

D283med and UW426 cells were seeded in six-well plates at a density of 2.5 × 10^5^ cells/well. After 24 hours of incubation, when the cells had reached approximately 70-80% confluency, they were infected with MV-NIS or MV-GFP at MOIs of 0.01, 0.1 and 1 in 200μl of OptiMEM. The virus was removed 2 hours later and replaced with 3 ml of DMEM. Cells were monitored under a microscope for the appearance of syncytia over the next 72 hours and photographed with a Spot RT KE/SE digital camera (Diagnostic Instruments Inc., Sterling Heights, MI). *In vitro* kill curves were constructed by determining the number of viable cells at each time point and MOI by trypan blue exclusion. The percentage of surviving cells was calculated by dividing the number of viable cells in an infected well by the number of viable cells in the uninfected well corresponding to the same time point. Sample and control wells were seeded and counted in triplicate.

### In vitro MV-NIS-mediated ^125^I uptake and retention assays

UW426 and D283med cells were seeded in 6-well plates at a density of 1.5 × 10^5^ cells/well and 5 × 10^5^ cells/well respectively. The cells were infected 24 hours later with MOI 0.1 MV-NIS or MV-GFP in 250 μl OptiMEM and allowed to incubate for an additional 2 hours. Following infection, the media was aspirated and replaced with DMEM until the time of the uptake assay. At each time point, the cells were washed once with warm Hanks’ Balanced Salt Solution (HBSS) and then placed in 900 μl HBSS+10mM HEPES, with or without 100 μM KClO_4_. One-hundred μl of ^125^I (1 × 10^5^ cpm total) was then added and the cells were incubated for 45 minutes at 37°C. The plates were then washed with cold HBSS+10mM HEPES, aspirated, and 1 ml of 1M NaOH was added to each well. After shaking for 15 minutes, the NaOH solution was removed and its radioactivity was quantified with a Cobra II gamma counter. Samples were set up and quantified in triplicate. Data is presented as counts per minute per 10^4^ cells.

Radioiodine retention was measured using a slight modification of the above protocol. Following the 45 minute exposure of ^125^I, the media covering the cells was collected and replaced with fresh HBSS+10mM HEPES every 3 minutes for a total of 30 minutes. Cells were then lysed and collected in 1M NaOH at the last timepoint. Total radioactivity at the beginning of efflux was calculated by adding the cpm of each supernatant to that of the lysed cells. Each sample was run in quadruplicate.

### In vivo xenograft studies

Localized and disseminated models of medulloblastoma were constructed as described previously [12,13]. In brief, 1x10^6^ D283med-Luc cells suspended in 7 μl PBS were implanted into the caudate nucleus (localized model) or right lateral ventricle (disseminated model) of 5-week-old Hsd:Athymic Nude-*Foxn1nu* mice (Harlan Laboratories, Indianapolis, IN). Bioluminescent imaging was conducted prior to initiating treatment in order to ensure that tumor burdens were roughly equivalent. Treatment with MV-NIS (2 × 10^5^ pfu/dose) or an equivalent volume of an OptiMEM vehicle control was initiated seven days post tumor implantation for the localized medulloblastoma mice or three days for the disseminated model mice. The mice placed into ^131^I treatment groups were switched to low-iodine diets and given daily IP injections of 5μg L-thyroxine one week prior to the administration of ^131^I. A single 37 MBq dose of ^131^I (Cardinal Radiopharmacy, Columbus, OH) was delivered by IP injection 24–72 hours post MV-NIS treatment. The animals were observed over the following weeks and euthanized if they became lethargic, displayed cachexia or exhibited hemiparesis or other motor impairment. All studies involving animals were approved by the Institutional Animal Care and Use Committee at The Research Institute at Nationwide Childrens’ Hospital (protocol number: AR08-00019).

### Bioluminescent imaging of tumor and ^131^I uptake

Bioluminescent imaging was conducted using the Xenogen Ivis Spectrum (Caliper Life Sciences, Hopkinton, MA). Animals were given an IP injection of 4.5 μg Xenolight Rediject D-Luciferin (Caliper Life Sciences) and kept under general anesthesia with isoflurane in O_2_ delivered by a veterinary vaporizer. Images were obtained 20 minutes after luciferin administration. Uptake of ^131^I was visualized as Cherenkov luminescence on the same system [18]. Imaging of ^131^I uptake was performed 24 hours after tumor bioluminescent images were acquired to allow for complete clearance of the luciferin. Image acquisition time was set for a 5 minute exposure. Flux is displayed as average radiance (photons/second/cm^2^/steradian).

### Histopathological evaluation

At the time of necropsy, brains and decalcified spinal columns were fixed overnight in 10% buffered formalin phosphate. They were then paraffin embedded, cut into 4 μm tissue sections, and stained with hematoxylin and eosin (H&E). Individual sections were visualized under a Zeiss Axioskop 2 Plus microscope and photographed with a Zeiss AxioCam MRc camera (Carl Zeiss MicroImaging, LLC., Thornwood, NY).

### Immunohostochemistry

IHC of tissue slides with anti-Measles Nucleoprotein antibody (NB100-1856; Novus Biologicals, Littleton, CO) was carried out as described previously [13].

### Statistical analysis

Survival curves were generated using the Kaplan-Meier method and GraphPad Prism version 5.01 software (GraphPad Software, Inc.).Comparisons of survival were done via the log-rank test. Differences were considered statistically significant if p ≤ 0.05.

## Results

### MV-NIS infects medulloblastoma cells and promotes the uptake of radioiodine

**Figure 1:**
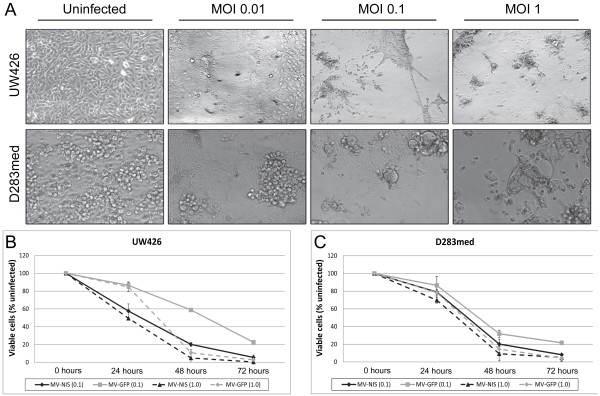
**MV-NIS induces syncytia formation and cell death in medulloblastoma cell lines. A**. UW426 and D283med medulloblastoma cell lines were infected with MV-NIS at MOIs of 0.01, 0.1 and 1 and then monitored for the appearance of syncytia formation over the next three days. The photographs shown here were taken at 100X magnification after 48 hours of infection. **B-C**. *In vitro* kill curves for UW426 and D283med infected with MV-NIS or MV-GFP. Viability was determined by trypan blue exclusion, and each sample was run in triplicate. The number of viable cells were averaged and expressed as a percentage of an uninfected control for each corresponding timepoint. Error bars represent one standard deviation.

**Figure 2:**
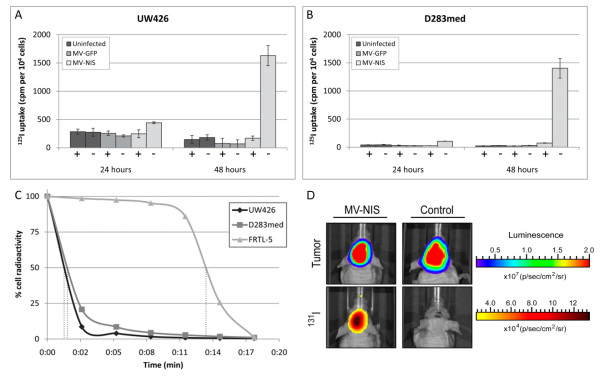
**MV-NIS promotes radioiodine uptake in infected medulloblastoma cells.** Radionuclide uptake assays were performed with **A**. UW426 and **B**. D283med. Each cell line was infected with MV-NIS or MV-GFP (MOI of 0.1) and then exposed to 1 × 10^5^ cpm of ^125^I at 24, 48 or 72 hours post infection. Due to excessive cell death at 72 hours, only data from the 24 and 48 hour timepoints are shown. The addition of KClO_4_ to the cells effectively blocked incorporation of ^125^I. Each sample was run in triplicate, with error bars representing one standard deviation. +/− denotes the presence or absence of KClO_4_ respectively. **C**. Radioiodine kinetics in UW426 and D283med 48 hours after MV-NIS infection. Efflux of ^125^I is rapid, with a t_1/2_ retention time of approximately 1.5 minutes for each cell line. Data points are the average of four independent samples. **D**. Medulloblastoma xenografts incorporate ^131^I following MV-NIS infection. The mouse in the left panels received a 2 × 10^5^ TCID_50_ intratumoral injection of MV-NIS three days following tumor implantation whereas the mouse in the right panels was given an equal volume of OptiMEM to serve as a vehicle control. Tumor bioluminescence was visualized 48 hours later, and the mice were subsequently given an IP injection of 37 MBq ^131^I. Cherenkov luminescence from the irradiated tumors was then visualized the following day.

We initially tested the efficacy of the MV-NIS virus *in vitro* against two established medulloblastoma cell lines, UW426 and D283med (Figure 1). These cell lines were previously shown to express abundant levels of the measles virus receptor CD46 and were susceptible to infection with an attenuated Edmonston-strain MV [12]. The addition of the NIS gene had no impact on measles virus’ cytotoxic activity, and syncytia formation was readily observed in both UW426 and D283med infected at MOIs as low as 0.01 within 48 hours (Figure 1A). *In vitro* kill curves showed that MV-NIS was just as effective or better at killing these medulloblastoma cell lines than a control MV encoding green fluorescent protein (MV-GFP), resulting in greater than 90% cell death within 72 hours of infection (Figure 1B-C). We confirmed the expression of functional NIS by performing radioiodide ^125^I uptake assays in UW426 and D283med following 24, 48 or 72 hours of infection with MV-NIS or MV-GFP. Increased uptake of ^125^I was observed only in the cells infected with MV-NIS, peaking at 48 hours post infection (Figure 2A-B). Extensive cell-death prevented measurement of ^125^I uptake after 72 hours of infection. The addition of KClO_4_, a competitive substrate for NIS, resulted in the near complete elimination of ^125^I uptake. The failure of MV-GFP infected cells to incorporate ^125^I demonstrates that NIS expression is responsible for the observed increases in ^125^I uptake. We then performed radioiodide retention studies in order to gauge how long the MV-NIS infected cells were retaining ^125^I. For these experiments, we infected UW426 and D283med with MV-NIS (MOI 0.1) for 48 hours and incubated them in the presence of ^125^I. The media covering these cells was then collected and replaced at 3 minute intervals, and the radioactivity in each sample was measured at the experiment endpoint. A rat thyroid cell line, FRTL-5, was similarly assayed for the purpose of comparison. We found the efflux of ^125^I to be rapid and comparable for each medulloblastoma cell line, displaying a ^125^I t_1/2_ retention time of approximately 1.5 minutes. In contrast, the t_1/2_ retention time in FRTL-5 was approximately 13.5 minutes (Figure 2C).

In order to determine whether MV-NIS infection could also promote radioiodine uptake in our *in vivo* model of localized medulloblastoma, we treated mice bearing D283med-Luc tumors with MV-NIS (2 × 10^5^ TCID_50_) and gave them a single IP injection of ^131^I (37 MBq) 48 hours later. The mice were imaged the following day with a Xenogen Ivis Spectrum imaging system. As a β^-^ particle emitter, ^131^I generates Cherenkov radiation as it decays, producing visible light that can be detected with ultra-sensitive charge-coupled device cameras [19,20]. Figure 2D shows representative bioluminescent images of an MV-NIS treated mouse side by side with a vehicle control. The MV-NIS mouse shows an increased bioluminescent signal originating from the tumor where ^131^I has accumulated. Strong bioluminescent signals were also noted in the stomach and bladder regions of some of the mice (data not shown).

We conducted similar studies with mice implanted with D283med-Luc in their lateral ventricles. In this particular model, the tumor disseminates within the CSF into the cranial and spinal subarachnoid spaces and closely recapitulates human disseminated medulloblastoma [13]. Despite repeated efforts, we were unable to detect ^131^I accumulation in the spinal tumors of these mice (data not shown).

### MV-NIS treatment with and without 131I prolongs survival in mouse xenografts

**Figure 3:**
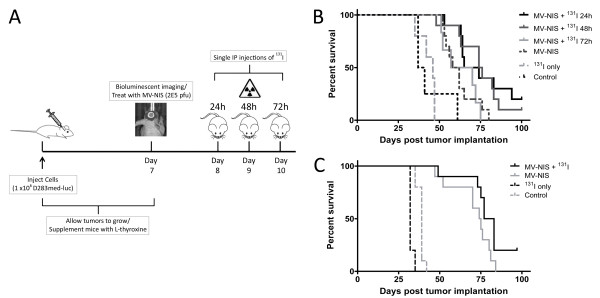
**MV-NIS treatment with and without **^**131 **^**I prolongs survival in mouse models of medulloblastoma. A**. Experimental design of the localized medulloblastoma MV-NIS + ^131^I survival study. **B**. Kaplan-Meier survival analysis of mice with localized medulloblastoma tumors treated with 2 × 10^5^ TCID_50_ MV-NIS seven days post tumor implantation. The mice were subsequently given a 37 MBq dose of ^131^I by IP injection at 24, 48, or 72 hours following MV-NIS treatment. The ^131^I-only mice were given radioiodine five days following tumor implantation. Mice denoted as controls were treated with OptiMEM. **C**. Survival analysis of mice with disseminated tumors. MV-NIS treatment was administered three days after tumor implantation

We next sought to determine whether MV-NIS virotherapy in combination with ^131^I conferred any survival advantage over MV-NIS alone in mice bearing intracranial medulloblastoma tumors. A total of 48 mice were implanted with D283med-luc cells in their caudate nuclei, and the tumors were given seven days to establish prior to treatment. During this time, the animals were switched to a low-iodine diet and given daily IP injections of L-thyroxine in order to suppress their thyroidal expression of NIS [21]. We imaged the tumors 7 days following implantation and observed that the animals had comparable tumor burdens on the basis of total emitted flux, however 4 mice had developed spinal metastases and were excluded from further study. The remaining mice were randomly assigned into the following groups: MV-NIS only (5 mice); MV-NIS + ^131^I at 24 hours (10 mice); MV-NIS + ^131^I at 48 hours (10 mice); MV-NIS + ^131^I at 72 hours (6 mice); ^131^I only (5 mice); and vehicle control (8 mice). Mice in the treated groups were given a single intratumoral injection of MV-NIS (2 x 10^5^ TCID_50_), followed by an IP injection of ^131^I at the appropriate timepoint. A schematic of the experimental design is shown in Figure 3A. All MV-NIS treated groups exhibited significant increases in survival time compared to the vehicle and ^131^I controls (p < 0.0001) (Figure 3B). Mice that received ^131^I at 24 or 48 hours after MV-NIS treatment, however, displayed statistically significant prolongation of survival compared to those given MV-NIS alone (p = 0.01 and 0.009 respectively). One of the mice in the 24 hour group and one in the 48 hour group survived symptom-free until the experiment endpoint at 100 days post tumor implantation. In contrast, the mice given ^131^I after 72 hours exhibited similar survival times to those given MV-NIS alone (p = 0.3). The administration of ^131^I by itself without prior MV-NIS treatment was found to produce no survival benefit over the vehicle controls (p = 0.3).

A similar series of survival studies was conducted in 35 mice with disseminated D283med-luc tumors (Figure 3C). In contrast to the localized medulloblastoma model, the mice in this set of experiments were treated 3 days after tumor implantation as opposed to 7 days. This discrepancy in timing was necessary as rapid occlusion of the ventricles by growing tumor cells can prevent efficient spread of the virus to distant sites [13]. Twenty mice were treated with MV-NIS, and 10 of these animals were subsequently given an IP injection of ^131^I 48 hours afterwards. The remaining 15 mice served as vehicle and ^131^I controls (n of 10 and 5, respectively). MV-NIS treatment had a significant impact on overall survival (p < 0.0001) and nearly doubled median survival times from 39 days for the control animals to 74.5 days and 80 days for the MV-NIS and MV-NIS + ^131^I animals respectively. The addition of ^131^I to MV-NIS only produced a trend towards increased survival over the virus alone however, and did not quite reach statistical significance (p = 0.06). Two mice from the MV-NIS + ^131^I group survived symptom-free until the experiment endpoint at 100 days post tumor implantation, whereas the entirety of the MV-NIS only group succumbed within 84 days.

### Histology of MV-NIS treated tumors

**Figure 4:**
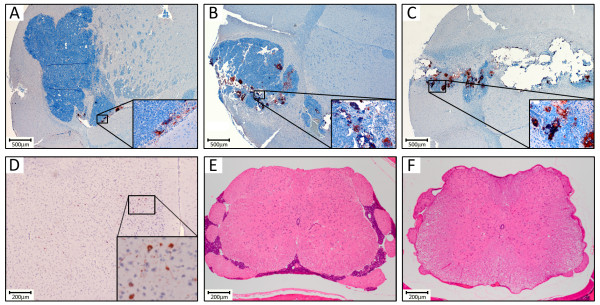
**Histological examination of mice treated with MV-NIS.** Representative IHC of a mouse brain after staining with an anti-MV nucleoprotein antibody **A**. 7 days, **B**. 14 days and **C**. 21 days following intratumoral injection with 2 × 10^5^ TCID_50_ MV-NIS. **D**. IHC of a tumor-free mouse brain with the same antibody, showing sparse off-target infection of cortical neurons. **E**. H&E staining of a spinal cord section from a control mouse in the disseminated medulloblastoma survival study showing extensive tumor infiltration. **F**. A spinal cord section from a mouse treated with MV-NIS + ^131^I. No evidence of tumor could be found in the spinal canals of five of six total animals in this treatment group.

Distribution of MV-NIS in the treated tumors was evaluated by immunohistochemistry (IHC) using an antibody specific for the MV nucleoprotein. Mice bearing D283med-luc intracranial tumors were treated once with MV-NIS (2 × 10^5^ TCID_50_) and then sacrificed for analysis at days 7, 14 and 21 post treatment (Figure 4A-C respectively). Increasing numbers of syncytia and MV-NIS-positive tumor cells could be detected at each timepoint, concomitant with greater levels of tumor clearance. When we performed histological examination of the animals in the localized medulloblastoma model survival studies, we found substantial tumor masses in the caudate nuclei of the control and ^131^I-only mice. Brains from all groups of MV-NIS treated mice showed large areas of tumor clearance surrounding the injection site, but small foci of tumor that had escaped MV oncolysis could be detected at distant sites of the cerebellum and brainstem. Conversely, the animals that survived free of visible symptoms until the experiment endpoint were also found to be free of viable tumors (data not shown).

During the course of this examination, we noted that several additional treated animals that had succumbed prior to the experiment endpoint were also determined to be tumor-free. These included two additional animals in the MV-NIS with ^131^I at 24 hours group (three of 10 animals tumor-free), four animals from the MV-NIS + ^131^I at 48 hours group (five of 10 animals tumor-free), and three animals from the MV-NIS + ^131^I at 72 hours group (three of six animals tumor-free). Prior to death, these animals had exhibited significant wasting, became lethargic and had assumed a kyphotic posture. Because these symptoms have been associated with MV-induced encephalitis in immunocompromised mice [22], we performed IHC on brain sections from these seemingly cured animals to assess the extent of any residual MV infection (Figure 4D). Although off-target infection of cortical neurons was evident in some of these sections, these events were generally rare and unaccompanied by any overt indications of an inflammatory response that would be indicative of encephalitis.

We also performed histological examination of the brains and spinal cords from mice in the disseminated medulloblastoma model survival studies. Tumor deposits could be found in the ventricles, cerebellum, brainstem, and cranial and spinal subarachnoid spaces of the OptiMEM control and ^131^I-only mice (Figure 4E). Comparable tumor masses were also found in the MV-NIS treated groups, suggesting that pockets of the tumor had managed to escape MV oncolysis or radiological cell killing. We could find no evidence of spinal metastases in two of the six mice from the MV-NIS only group and five of the six mice from the MV-NIS + ^131^I group however, despite the presence of these tumors being confirmed via bioluminescent imaging at the outset of the experiment (Figure 4F).

## Discussion

Although current treatment strategies for medulloblastoma are effective, they carry inherent risks and are associated with significant morbidity [23-25]. Radiation therapy, in particular, is known to produce a broad spectrum of cognitive and endocrine impairments in surviving patients [26,27]. With these issues in mind, the focus of many labs has shifted towards identifying therapies that are both effective against and highly specific for transformed cells, hopefully mitigating the need for more conventional therapy. In this study, we evaluated the oncolytic activity of MV-NIS used in conjunction with ^131^I against two xenograft models of medulloblastoma, seeking to combine measles virotherapy with targeted radiotherapy. As a derivative of the attenuated Edmonston vaccine strain, MV-NIS is able to efficiently enter cells through the CD46 receptor and promote mass cell-cell fusion and death via apoptosis [8,9]. Successful infection and viral propagation is dependent upon high expression of CD46 on the target cell membrane, and this reliance on receptor abundance allows MV-NIS to functionally discriminate between normal and tumor cells [28,29]. The CD46 receptor is highly expressed in medulloblastoma, making this cancer a suitable target for MV virotherapy [12].

MV-NIS has shown impressive oncolytic activity in multiple preclinical tumor models [14,28,30-32], and its ability to promote iodide uptake in infected tumor cells has made targeted radiotherapy feasible for cancers of various origins. Targeted radiotherapy differs from conventional external beam radiotherapy in that it delivers low doses of radiation over prolonged periods of time and tends to promote non-necrotic mechanisms of cell death through localized, but potent, bystander effects that minimize unintended damage to the normal surrounding tissue [33]. Additional cell killing by radiological cross-fire is also predicted to occur with radionuclides like ^131^I, whose decay produces β^-^ particles that travel long distances (up to 0.36 mm) and introduce single-strand DNA breaks in the cells they traverse before dissipating [34]. Since medulloblastomas are highly susceptible to MV oncolysis and known to be radiosensitive [2,35], we hypothesized that a targeted radiovirotherapy approach using MV-NIS with ^131^I would be more effective than MV virotherapy alone.

We were able to confirm that a single intratumoral injection of MV-NIS was capable of promoting ^131^I uptake in the cranial tumors of treated mice using an emerging imaging modality known as Cherenkov luminescent imaging (CLI). Cherenkov radiation is a phenomenon where charged particles move faster than the speed of light through the medium in which they travel, emitting optical photons in the process [18]. These particles, which are produced during the decay of β^-^-emitting radionuclides, can be subsequently detected by a sensitive charge-coupled device camera like the Xenogen Ivis Spectrum used in the experiments detailed above. Although the utility of CLI as an imaging modality is currently limited to small-animal molecular imaging, it can provide a means to semi-quantitatively determine signal intensity and spatial distribution of radionuclides like ^131^I where conventional imaging modalities such as positron emission tomography (PET) or single-photon emission computed tomography (SPECT) are unavailable or cost prohibitive [36].

Kaplan-Meier analysis of our localized medulloblastoma survival studies suggests that the animals did benefit from the inclusion of ^131^I to their MV-NIS treatment, provided that the ^131^I was administered within a 24–48 hour window after infection. These time points most likely encompass the period of peak MV-NIS infection in our medulloblastoma model, where infected tumor cells have begun expressing NIS but have yet to fully undergo lysis, and would correlate with our *in vitro* observations (Figure 2A-B). The addition of ^131^I at 72 hours post infection, the last time point we evaluated, had no impact on overall survival. These results are also in agreement with similar observations reported by Penheiter et al., who evaluated MV-NIS radiovirotherapy in a mouse xenograft model of pancreatic cancer [32].

In our disseminated medulloblastoma survival studies, the addition of ^131^I to MV-NIS treated animals produced a moderate increase in survival over MV-NIS only treated animals that approached the threshold of statistical significance (p = 0.06). Disseminated disease is an especially grave prognostic factor, and has proven to be exceptionally difficult to effectively treat [13]. Despite an overt lack of synergy with ^131^I, MV-NIS was effective as an oncolytic agent and extended the median survival times of the treated animals to nearly double that of their respective vehicle controls (Figure 3C). It is also important to note that we were unable to find spinal tumors in five of the six animals given MV-NIS + ^131^I at time of autopsy, suggesting that these animals may have died due to residual tumors around their brainstems and cerebella. Future experiments aimed at optimizing virus delivery and ^131^I dosing may eventually yield enhanced efficacy in this model.

During the course of the localized medulloblastoma survival studies, a substantial number of treated and otherwise tumor-free mice succumbed prior to the experiment endpoint. Although it is difficult to draw definitive conclusions, there are two possible explanations for these unexpected occurrences. One possibility is that the mice developed an adverse reaction to the measles virus on account of their severely immunocompromised state. In mice, resistance and susceptibility to MV-induced encephalitis is governed by their major histocompatibility complex haplotype [37]. Nude mice, characterized by their thymic aplasia, are unable to produce functional CD4+ and CD8+ T lymphocytes, which play important roles in clearing MV from the central nervous system [38]. Neurologic disease has been shown to occur in these animals following intracerebral inoculation with Edmonston strain MV, albeit after long incubation periods (49 to 140 days after 10^4^ pfu virus) [22]. For comparison, the seemingly cured mice from our studies died between days 45 and 87 following MV-NIS treatment. We did observe off-target MV infection in a small population of neurons from the treated mice, but these were unaccompanied by signs of inflammation, neuronal death, or any symptoms of neurotoxicity in the animals prior to their sacrifice. An alternative explanation may be that the animals succumbed to the effects of gastrointestinal toxicity following ^131^I administration. Although NIS is predominately expressed by thyroid follicular cells, it is also abundant in the gastric mucosa of mice [39]. Uptake of ^131^I by these gastric cells will eventually lead to their death, culminating in animal malnutrition over the long term [15,39]. Incidentally, the mice in our studies that died free of tumor all came from treatment groups that received an IP dose of ^131^I, but we did not collect their gastric tissue for further analysis at the time of this study. A recently published paper by Opyrchal and colleagues examined MV-NIS + ^131^I radiovirotherapy in glioma however, and determined gastrointestinal toxicity to be the likely cause of premature death in their animals under study [15].

Whether the cause of death in these animals was due to the MV or the ^131^I itself, the toxicity observed here should not extend to immunocompetent human beings. The side effects for ^131^I-based therapies in humans are generally mild when administered in reasonable doses [40,41] and the safety of MV-NIS has been vetted in preclinical toxicity studies and phase I clinical trials [42-44]. MV-NIS is a derivative of the Edmonston vaccine strain, a highly attenuated strain of measles virus that has a remarkable safety record spanning over a billion recipients worldwide [45]. With close to five decades of use, its reversion to pathogenicity has never been reported [46]. Extensive studies have also shown no clinical evidence of toxicity in non-human primates following intracerebral injection of Edmonston strain MV [47,48]. In addition, phase I clinical trials with oncolytic MV are presently underway for treatment of multiple myeloma [44], recurrent glioblastoma multiforme [49], and ovarian cancer [43]. While data from these trials is still forthcoming, no dose-limiting toxicity has been observed following delivery of MV up to 10^9^ TCID_50_ by IP or IV administration and up to 10^7^ TCID_50_ for MV delivered through the central nervous system [50]. We have recently proposed a Phase 1 clinical trial investigating the use of oncolytic MV to treat recurrent medulloblastoma. While we foresee no complications with MV-associated toxicity, additional measures to further restrict MV replication to tumor cells are available should they be deemed necessary [51,52].

## Conclusions

The data presented here show that MV-NIS virotherapy is an effective means of treating medulloblastoma in mouse xenografts, and that its oncolytic activity against localized tumors can be further enhanced by the subsequent IP administration of 37 MBq of ^131^I at 24 or 48 hours of viral delivery. Proper timing of ^131^I administration treatment appears to be critical in this tumor model, as this survival benefit was lost when ^131^I was given 72 hours after MV-NIS treatment. Significant questions remain to be addressed however. Despite its utility in determining whether ^131^I has been concentrated by the tumor, CLI currently lacks the resolution to provide tomographic detail about and quantification of radioactivity uptake. Understanding these parameters will be necessary before extrapolating the potential of MV-NIS-based therapies to human medulloblastoma patients, as effective radionuclide therapy is dependent upon total isotope uptake and retention in order to deposit therapeutically relevant levels of energy in the tumor [53]. Our *in vitro*^125^I efflux experiments suggest that radioiodine retention in medulloblastoma cells is fleeting (Figure 2C), so additional measures to improve iodide organification or slow its release may be necessary in order to achieve clinical benefit. Certain drugs, such as 17-(allylamino)-17-demethoxygeldanamycin and 4,4’-diisothiocyanatostilbene-2,2’-disulfonic acid, have been shown to increase intracellular iodide retention times in thyroid cancer cells [16], and it is possible that they may exert a similar effect on NIS-expressing medulloblastoma cells. Future studies using MV-NIS to treat medulloblastoma should also be aimed at ascertaining optimal viral and ^131^I dosing rates and expanded to include non-invasive monitoring of viral propagation and distribution. Our initial findings are encouraging, however, and suggest that MV-NIS mediated radiovirotherapy may have clinical utility in the treatment of medulloblastoma.

## Competing interests

The authors declare that they have no competing interests.

## Authors’ contributions

BH, AWS and CR participated in the design of the study, conducted the in vitro and in vivo experiments, and drafted the manuscript. CRR performed the histological analysis and helped analyze the data. SJR and EG provided the MV-NIS virus, technical expertise and contributed to the experimental design of the study. All authors read and approved the final manuscript.

## Pre-publication history

The pre-publication history for this paper can be accessed here:

http://www.biomedcentral.com/1471-2407/12/508/prepub
